# Up-Regulated MicroRNA499a by Hepatitis B Virus Induced Hepatocellular Carcinogenesis via Targeting MAPK6

**DOI:** 10.1371/journal.pone.0111410

**Published:** 2014-10-23

**Authors:** Zheng Xiang, Sen Wang, Yao Xiang

**Affiliations:** 1 Chongqing Key Laboratory of Department of General Surgery, Chongqing Medical University, Chongqing, China; 2 Department of Gastrointestinal Surgery, First Affiliated Hospital of Chongqing Medical University, Chongqing, China; 3 Key Laboratory of Molecular Biology on Infectious Diseases, Chongqing Medical University, Chongqing, China; Taipei Medical University, Taiwan

## Abstract

Emerging evidence showed miR499a could not only function as an oncogene but also as a tumor suppressor in various types of cancer, such as melanoma. However, whether miR499a was involved in hepatocarcinogenesis remains unknown. We previously reported that miR499a was up-regulated in HBV-mediated hepatocellular carcinoma (HCC). In this study, we found that HBV could induce the expression of miR499a by promoting its promoter activity. In addition, we reported that miR499a increased cell proliferation and cell migration of HCC cells. MAPK6 was further identified as a target of miR499a, which could also be down-regulated by HBV. Moreover, we demonstrated that MAPK6 could rescue the cell growth induced by miR499a and HBV. These findings indicated that miR499a might play an oncogene role by targeting MAPK6 in the development and progression of HBV-related HCC.

## Introduction

Chronic HBV infection, which is endemic in such as South-East Asia and Sub-Saharan Africa, are greatly detrimental to human health and affect quality of life [1], [2]. Numerous studies have shown that hepatocellular carcinoma (HCC) is closely associated with Hepatitis B virus (HBV) infection [3]. However, the pathogenic mechanism of HBV-inducing HCC remains elusive.

MicroRNAs (miRNAs), one of short non-coding RNAs, play a pivotal role in negatively repression of gene expression by interacting with the 3′UTR of protein-coding mRNA. Emerging evidence has demonstrated that nearly all major biological and cellular events were regulated by miRNAs [4]–[8]. The expression of miRNA also plays an important role in the carcinogenesis of HBV-induced HCC [9]. Several research found that miR499a could increase the risk of death in variety of diseases, such as acute non-ST elevation myocardial infarction, colorectal cancer and non-small cell lung cancer [10]–[12]. Li *et al*. found that miR499a regulated cell proliferation and apoptosis during late-stage cardiac differentiation [13]. Our previous research (miRNA microarray) has shown that miR499a was up-regulated in HBV-related HCC cells. Whether miR499a was involved in the carcinogenesis and development of HBV-related HCC was unclear.

Mitogen-activated protein kinase(MAPK6, ERK3), a distinct MAPK subfamily, is one of atypical MAPK [14]. So far the only substrate of MAPK6 that has been identified is the MAP kinase-activated protein kinase MK5 [15], [16]. Numerous observations announced an involvement of the ERK3/4-MK5 pathway in cell cycle regulation. Meanwhile, a recent study reported that the ERK3/4-MK5 pathway play roles in tumor promoting and suppressing [17]. Then, what's the role of MAPK6 in the process of HCC?

In this study, we mainly explored the functions of miR499a in HCC cells, as well as the molecular mechanism of miR499a inducing HCC. Firstly, the expression levels of miR499a in HBV-related HCC cell lines were examined and the functions of miR499a were also analyzed. In addition, we explored the target gene of miR499a to disclose the underlying mechanism of miR499a functions in HCC. Our study will provide a novel pathogenic mechanism of miR499a contributing to HCC development.

## Result

### HBV up-regulated miR499a by increasing its promoter activity

**Figure 1:**
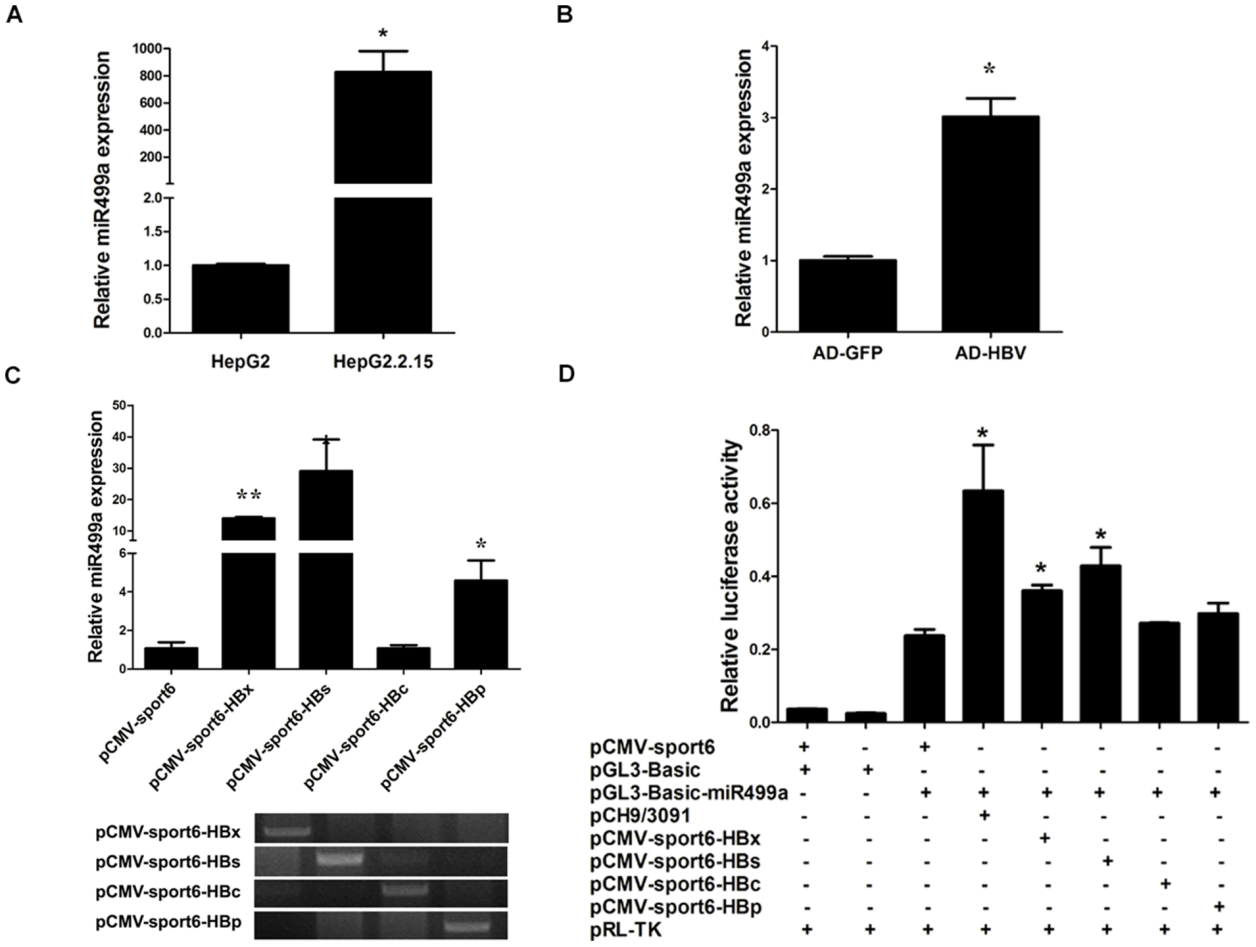
HBV up-regulated miR499a by increasing its promoter activity. (A) The relative expressions of miR499a in HepG2.2.15 and HepG2 cell lines were detected by qRT-PCR. (B) The expressions of miR499a in HepG2 cells infected with HBV adenovirus or GFP adenovirus were examined by qRT-PCR. (C) The expressions of miR499a in SMMC-7721 cells transfected with four major HBV protein vector were measured by qRT-PCR. And the results of RT-PCR showed that HBs, HBx, HBc and HBp could be expressed. (D) Luciferase reporter gene assay showed HBV, HBs and HBx could increase the activity of the miR499a promoter. miRNA abundance was normalized to U6 RNA. Empty pCMV-sport6 plasmid was used as a negative control. Statistically significant differences, arbitrarily set to 1.0, are indicated: *p<0.05, **p<0.01, Student's *t* test.

Firstly, miR499a expression in HepG2.2.15 and HepG2 cells were analyzed by quantitative real-time PCR. Consistent with our previous miRNA microarray result, miR499a expression was significantly up-regulated in HepG2.2.15 compared with HepG2 cells (Fig. 1A). MiR499a expression was also examined in Ad-HBV or Ad-GFP infected HepG2 cells. qRT-PCR result showed that the expression of miR499a was increased in Ad-HBV infected HepG2 cells in comparison with the control (Fig. 1B). Meanwhile, miR499a expression was upregulated by all the four major HBV proteins, especially by HBx and HBs (Fig. 1C).

To explore the molecular mechanism of HBV regulating miR499a expression, the miR499a promoter plasmid was respectively cotransfected with pCH9/3091 plasmid or the four major HBV proteins expression plasmids (pCMV-Sport6-HBx, pCMV-Sport6-HBs, pCMV-Sport6-HBc and pCMV-Sport6-HBp). The luciferase assay illuminated that HBV and two of its proteins (HBx and HBs) could markedly increase miR499a promoter activity (Fig. 1D). Together, these data demonstrated that HBV could up-regulate miR499a expression by increasing its promoter activity.

### MiR499a accelerated growth of HCC cells

**Figure 2:**
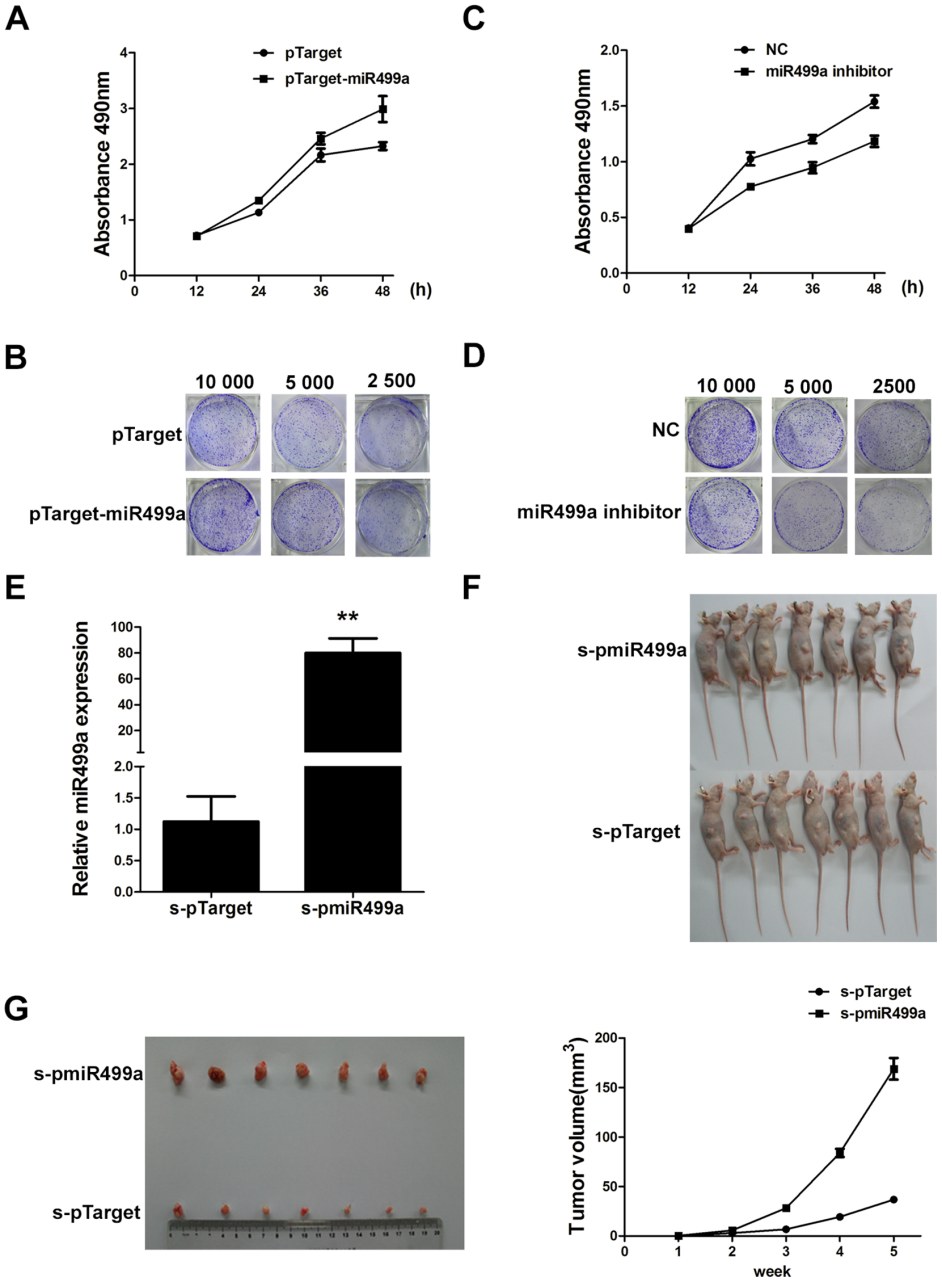
MiR499a accelerated growth of HCC cells. (A and B) Cell proliferation was measured by MTS and colony formation assay after SMMC-7721 cells transfected by pTarger or pTarget-miR499a. (C and D) MTS assay and colony formation assay were used to measure cell proliferation of SMMC-7721 cells transfected with miR499a inhibitor or its negative control. (E) The expressions of miR499a in stable cell line s-pTarget and s-pmiR499a were measured by qRT-PCR (*p<0.05, **p<0.01). (F) Stable cell line s-pTarget and s-pmiR499a were injected subcutaneously into nude mice. After implantation 2 weeks, s-pmiR499a cells produced larger tumors than s-pTarget cells. (G)Subcutaneous tumors formed and the growth curve of tumor volumes was shown.

To explore whether miR499a could affect cell proliferation of HCC cells, SMMC-7721 cells were transiently transfected with pTarget-miR499a or miR499a inhibitor, and then cell growth was measured. Both MTS and clone formation assay indicated that miR499a overexpression promoted cell proliferation of HCC cells (Fig. 2A, B). In contrast, the inhibition of miR499a suppressed cell proliferation (Fig. 2C, D).

In order to further certify the role of miR499a in cell proliferation, the cells stably expressing miR499a (s-pmiR499a) or control cells expressing vector (s-pTarget) (Fig. 2E) were injected subcutaneously into the dorsal flank of nude mice. The tumor became palpable after implantation 7 days and all the mice developed tumors at the end of the experiment (Fig. 2F). The tumor formed by miR499a-expressing cells showed increased tumor size and weight compared to control cells (Fig. 2G). These results suggested that miR499a could promote cell proliferation in vivo.

### MiR499a promoted migration of hepatoma cells

**Figure 3:**
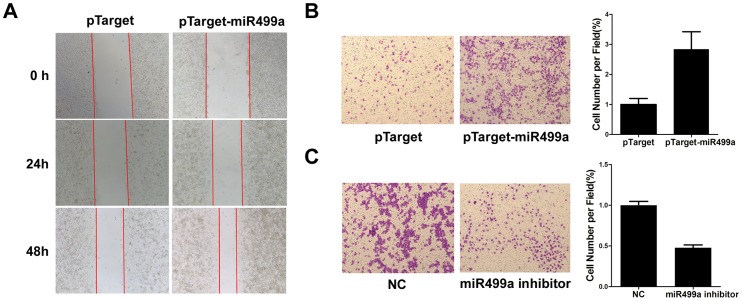
MiR499a promoted migration of hepatoma cells. (A) Wound healing assay was performed on SMMC-7721 cells transfected with pTarget or pTarget-miR499a (1×10^6^ per well, 6-well plates) with serum starvation. Red arrows noted the wound edge. (B) Representative images of transwell migration of SMMC-7721 cells transfected by pTarget-miR499a (left, magnification ×200). The mean number of migrated cells in three randomly selected fields were counted under the microscope were shown in bar graph (right). (C) The effects of down-regulated miR499a by transfected miR499a inhibitor were represented by transwell assay.

To determine whether miR499a was associated with migration of hepatoma cells, SMMC-7721 cells were transfected with pTarget or pTarget-miR499a and then wound healing assay was performed. As shown in Fig. 3A, miR499a overexpression group revealed an obvious increase in the cell migration ability relative to the control group. The result of transwell assay was in accordance with the result of wound healing assay (Fig. 3B). In contrast, the inhibition of miR499a decreased cell migration ability (Fig. 3C). These data indicated that miR499a could promote migration of hepatoma cells.

### MAPK6 was a target gene of miR499a

**Figure 4:**
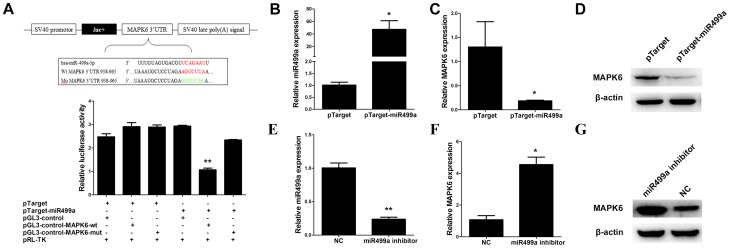
MAPK6 was the target gene of miR499a. (A) pGL3-control-MAPK6 plasmid contained a part of 3′UTR of MAPK6 sequence containing putative miR499a binding site. And pGL3-control-MAPK6-mut was mutanted the putative miR499a binding site. Luciferase reporter assays in SMMC-7721 cells, with cotransfection of pGL3-control-MAPK6 or pGL3-control-MAPK6-mut and miR499a as indicated. (B) qRT-PCR results showed the expression level of miR499a in SMMC-7721 cells transfected with pTarget or pTarger-miR499a. (C and D) qRT-PCR and Western blot were performed to analyze mRNA and protein levels of MAPK6 in SMMC-7721 cells transfected with pTarget or pTarger-miR499a. (E) The expression level of miR499a in SMMC-7721 cells transfected with miR499a inhibitor was detected by qRT-PCR. (F and G) The mRNA and protein levels of MAPK6 were indicated by qRT-PCR and Western blot in SMMC-7721 cells after transfection of miR499a inhibitor.

To determine putative miR499a targets, TargetScan and MiRanda bioinformatics algorithms were used. Software analysis revealed that MAPK6 might be a potential target of miR499a based on putative target sequences of MAPK6 mRNA 3′UTR. Luciferase assay was performed to determine whether miR499a could directly target 3′UTR of MAPK6 mRNA in SMMC-7721 cells. The target sequence of MAPK6 3′UTR or mutant sequence was cloned into luciferase vector. Luciferase assay showed that miR449a suppressed the luciferase activity of 3′UTR of MAPK6. (Fig. 4A). In contrast, miR499a overexpression has no effect on luciferase activity of the mutant 3′UTR reporter (Fig. 4A).

Moreover, overexpression of miR499a significantly down-regulated MAPK6 expression at both mRNA and protein levels in SMMC-7721 cells (Fig. 4B, C and D). On the contrary, inhibition of miR499a decreased both mRNA and protein of MAPK6 expressions (Fig. 4E, F and G). Together, these results indicated that MAPK6 was a target of miR499a.

### HBV down-regulated the expression of MAPK6

**Figure 5:**
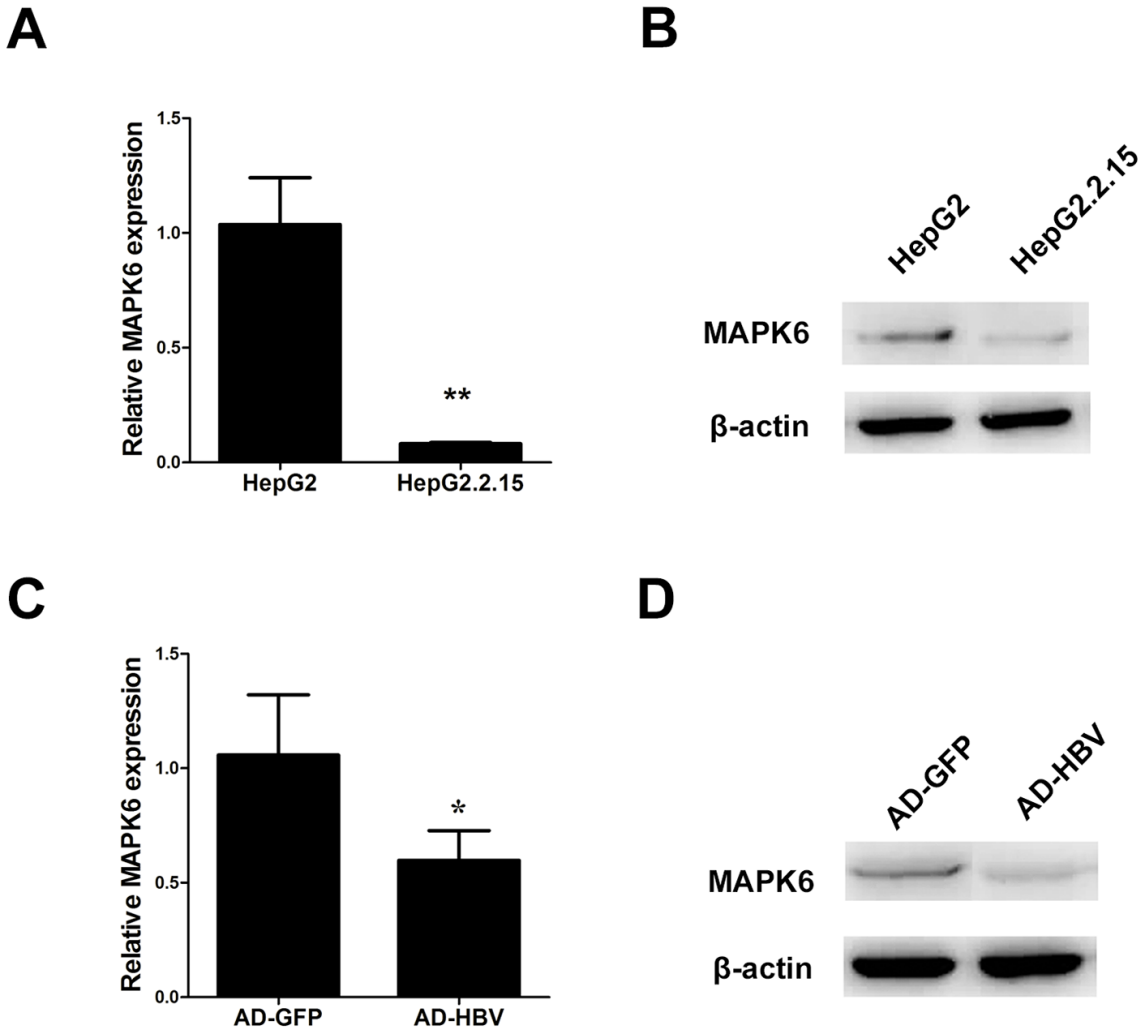
HBV down-regulated the expression of MAPK6. (A and B) qRT-PCR and Western blot analysis showed the mRNA and protein levels of MAPK6 in HepG2 cells and HepG2.2.15 cells. (C and D) The levels of MAPK6 mRNA and protein were tested in HepG2 infected with HBV adenovirus or GFP adenovirus. Statistically significant differences are indicated:*p<0.05, **p<0.01, Student's *t* test.

Above results prompted us to examine whether HBV could regulate MAPK6 expression. Firstly, the expressions of MAPK6 were measured in HepG2 and HepG2.2.15 cells. As shown in Fig. 5A and B, the expressions of MAPK6 in HepG2 cells were higher than HepG2.2.215 cells at both mRNA and protein levels. Meanwhile, MAPK6 expression was significantly decreased in Ad-HBV infected cells in comparison with its control group (Fig. 5C, D). These results revealed that HBV could inhibit MAPK6 expression.

### MiR499a promoted cell growth by reducing MAPK6

**Figure 6:**
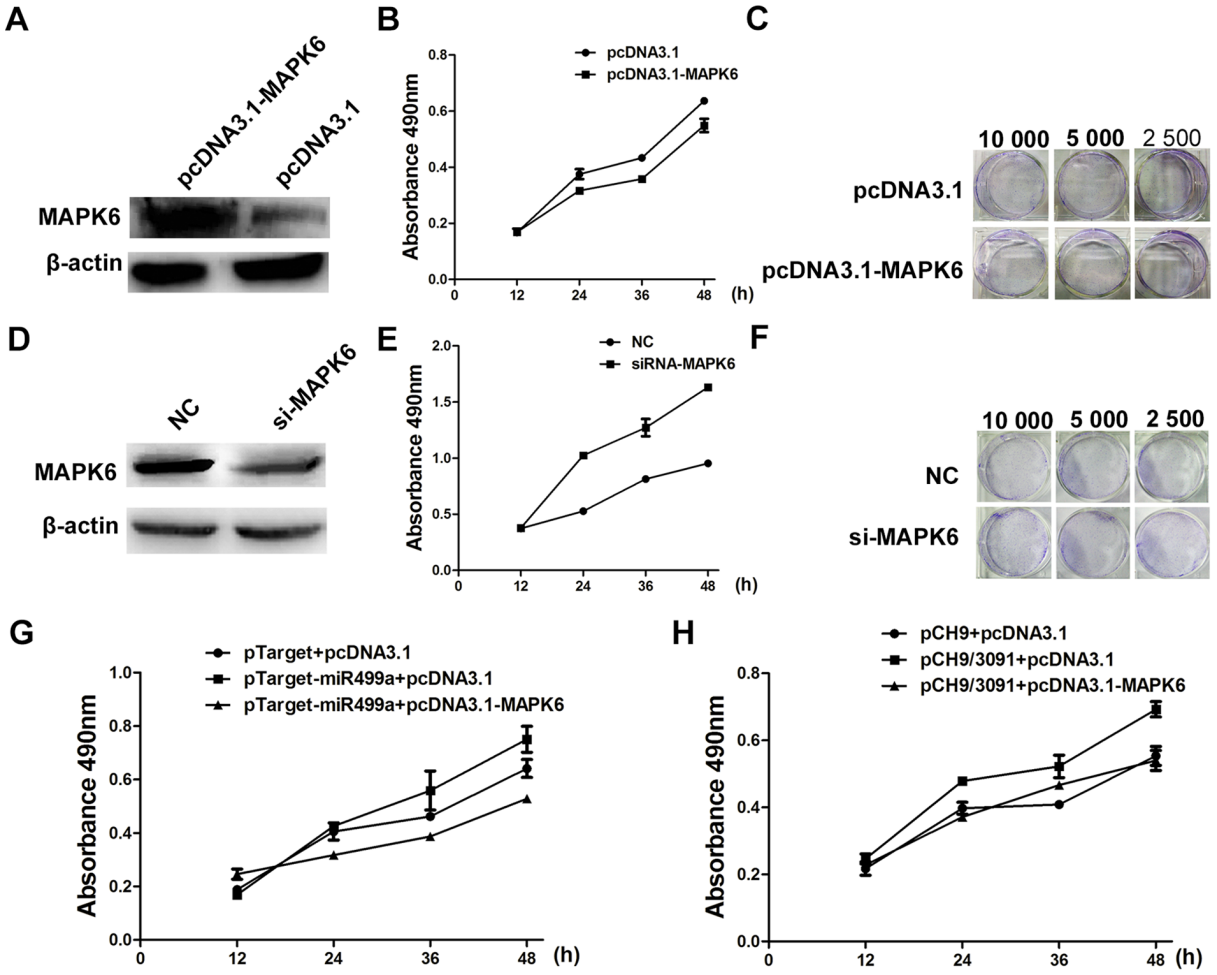
MiR499a promoted cell growth by reducing MAPK6. (A) Western blot analysis showed the protein level of MAPK6 in SMMC-7721 cells transfected with overexpression plasmid pcDNA3.1-MAPK6 or control plasmid pcDNA3.1. (B) Cell proliferation was measured after transfected by pcDNA3.1 or pcDNA3.1-MAPK6 by MTS assay. (C) The colony formation assay showed the effects of MAPK6 overexpression on cell proliferation in concentration gradient. (D) The expression of MAPK6 in SMMC-7721 cells transfected with NC or si-MAPK6 was detected by Western blot. (E) Proliferation of cells transfected with si-MAPK6 was obviously increased as compared with NC transfection by MTS assay. (F) Representative pictures of colony formation assay of si-MAPK6 transfected SMMC-7721 cells. (G) The results of MTS assay illustrated the increase of cell proliferation transfected with pTarget-miR499a could be reduced by the overexpression of MAPK6. (H) The increase of cell proliferation by the overexpression of miR499a mediated by HBV could be inhibited though the overexpression of MAPK6. The results were measured by MTS assay. Statistically significant differences are indicated:*p<0.05, **p<0.01, Student's *t* test.

Accumulating evidence have demonstrated that the overexpression of MAPK6 lead to inhibition of cell proliferation [19]. MTS and colony formation assay were performed to analyze the effect of MAPK6 on cell proliferation of HCC cells. As shown in Fig. 6A, B and C, cell proliferation was markedly inhibited when MAPK6 overexpressed. In contrast, both MTS and colony formation assay showed the inhibition of MAPK6 could obviously promote cell growth (Fig. 6D, E and F). Taken together, these data suggested that MAPK6 could suppress cell proliferation.

To explore whether miR499a promoted cell growth by targeting MAPK6, MTS was used to measure cell proliferation of SMMC-7721 cells cotransfected with pTarget-miR499a and pcDNA3.1-MAPK6. As shown in Fig. 6G, MAPK6 abolished the growth-promoting effect induced by miR499a. Furthermore, MAPK6 could also rescue HBV inducing cell growth (Fig. 6H). All the above data suggested that HBV or miR499a promoted proliferation of HCC cells probably through MAPK6.

## Discussion

It is estimated there are more than 387 million HBV carriers and HBV infected people in the world. HBV was widely known as a high risk factor of HCC [20], [21]. In recent years, numerous studies have shown that miRNA play important roles in organisms physiological and pathological processes. In particular, tumorigenesis was highly related with negatively regulation of genes expression by miRNA, such as chronic lymphocytic leukemia, lung cancer, breast cancer and colon cancer. Several reports revealed that HBV infection could also alter the host miRNAs expression [22], [23].

MiR499a were reported to be associated with variety of tumors [10], [24]. Li *et al.* have reported that miR499 regulated cell proliferation and apoptosis during late-stage cardiac differentiation via SOX6 and cyclinD1 [13]. However, the role of miR499a in HCC was not reported. So the main topic of our study is the influence of HBV on miR499a and the functions of miR499a in HCC. In our study, we firstly found HBV could up-regulate miR499a by promoting its promoter activity. In further research, our study firstly pointed out that miR499a could improve hepatoma cell proliferation *in vitro* and tumor growth *in vivo*, and revealed that miR499a could increase cell migration of hepatoma cells. Therefore, we suggested that miR499a might act as oncogene in HCC.

MAPK6 (ERK3) is an atypical member of the MAPK family, which can reduce cell proliferation through ERK3/ERK4-MK5 pathway [17]. In our research, we firstly demonstrated MAPK6 was a direct target gene of miR499a. Our results showed that miR499a increased cell proliferation via targeting MAPK6. The regulation of miRNA was a multi-targeted, multi-step and intricately network in cells. Therefore other genes might also be affected by miR499a and contribute to the increase of cell proliferation, which worth to be investigated.

Our results indicated HBV could up-regulate miR499a and promote cell growth partly by inhibiting MAPK6 expression. Meanwhile miR499a increases cell proliferation by down-regulating MAPK6 expression. These data suggested that HBV promoted cell proliferation, at least partly, by regulating miR499a and MAPK6 expression. However, whether HBV promoted the activity of miR499a promoter by modulated upstream transcription factor was not investigated. We found that miR499a could promote cell migration. However, MAPK6 had no effect on migration of HCC cells. So the mechanism of miR499a promote migration need further investigation.

In summary, our findings thus provided a new perspective in understanding both the pleiotropic nature of miR499a and its contribution to HCC development. All the results suggested that miR499a might function as an onco-miRNA in HBV-related HCC.

## Materials and Methods

### Cell culture and Cell transfection

SMMC-7721 cells (from American Type Culture Collection, USA) were grown in RPMI 1640 medium (Hyclone) with 10% fetal bovine serum (FBS, Gibco). HepG2 and HepG2.2.15 cells (from American Type Culture Collection, USA) were maintained in MEM/EBSS (Hyclone) with 10% FBS. All cells were cultured in a humidified incubator at 37°C in 5% CO_2_. Vector transfection was carried out with Lipofecatmine 2000 reagent (Invitrogen) according to the manufacturer's instructions. Transfected cells were harvested at 48 hours.

### Vector and Vector construction

pCH9/3091, the HBV expression plasmid, was constructed by Michael *et al*. (Heidelberg University, Germany) and donated by Dr Lan Lin (Southwest hospital Affiliated with the Third Military Medical University, China). The pCMV-Sport6 plasmid was obtained from ATCC (American Type Culture Collection, USA). The pCMV-Sport6-HBx, pCMV-Sport6-HBs, pCMV-Sport6-HBc, and pCMV-Sport6-HBp plasmid were previously constructed in our laboratory and their correct expressions were confirmed in HepG2 cells (data not shown). The pGL3-Basic, pGL3-control and pRL-TK plasmids were purchased from Invitrogen (USA). The pTarget plasmid was purchased from Promega (USA). Ad-HBV adenovirus and its control Ad-GFP adenovirus were constructed by our laboratory.

The miR499a promoter construct (pGL3-Basic-miR499a) was amplified with sequence (−950–+50) containing 50 bp miR499a precursor by using the following primers: forward (5′-AGGTACCACAGCTAGTGAGTGAGGAAG-3′); reverse (5′-TCTCGAGACTGCAAGTCTTAACAGCC-3′). pTarget-miR499a, miR499a expression plasmid, was constructed with fragment of miR499a precursor and the sequence was amplified by the following primers: forward (5′-ACGACTCGAGCCCCATCTTCCAGAAGTCAC-3′); reverse (5′-ATAAAGTC GACGCAGCGGACGAAGTGGGCT-3′). pGL3-control-MAPK6-wt was constructed by inserting part of the MAPK6 3′UTR sequence containing putative miR499a binding site and amplified by the following primers: forward (5′-CATCTAGACATGATACCAGCAGCAAC-3′); reverse (5′-AATCTAGAAGTACGTGGACAGCCTTA-3′). pGL3-control-MAPK6-mut was constructed by insert with the MAPK6 3′UTR with point mutations in the seed sequence, which was amplified from the wild vector. The primers used were forward (5′-ATGCTCCTAGAGCGTCAGATTGTGTTTATTTTT-3′); reverse (5′-TAAACAC AATCTGACGCTCTAGGA GCATTTTA-3′). The full-length MAPK6 expression vector was built from MAPK6 mRNA using the following primers: forward (5′-ACGGTACCAGGGTTTCAAAATGGCAGAG-3′); reverse (5′-TCTCGAGTTAGTTCAGATGTTTCAGAATGCTG-3′) and cloned into pcDNA3.1, which was designated as pcDNA3.1-MAPK6. pTarget, pCMV-sport6, pGL3-control, pGL3-Basic vectors were used as control. Ad-HBV adenovirus and its control Ad-GFP adenovirus were constructed by our laboratory as following steps: HBV 1.3 fold genome was ligated into shuttle vector pAdTrack-TO4, then pAdTrack-TO4-HBV1.3 was linearized by *Pme*I and transfected into BJ5183 cells containing pAd-Easy1 to form a recombinant plasmid. After that the confirmed recombinant plasmid was linearized by *Pac*I restriction endonuclease and transfected into HEK-293 cells to generate recombinant adenoviruses [18].

### RNA interference

MiR499a inhibitor, inhibitor negative control (NC), siRNA duplexes target MAPK6 were synthesized and purified by Invitrogen (shanghai, China). SiRNA duplexes with non-specific sequences were used as siRNA negative control (NC). Different siRNAs were transfected separately into cells by using Lipofecatmine 2000 reagent.

### Stable cell lines generation

The pTarget-miR499a plasmid or empty vector (pTarget) were transfected into the SMMC-7721 cells and selected with G418 (800 ug/ml) for 4 weeks. Then the stable cell lines called s-pmiR499a and s-pTarget were generated and cultured in medium with 400 µg/ml G418.

### RNA isolation, reverse transcription, and quantitative real-time PCR

Total RNA of the cells was extracted with Trizol reagent (Invitrogen). In order to quantitate miR499a expression, miRNA cDNA Kit (CWBIO, China) and miRNA Real-Time PCR Assay Kit (CWBIO, China) were used. U6 was used as a miRNA internal control. To measure the mRNA expression of MAPK6, total RNA was reversely transcribed using the Reverse Transcription System (Promega, Madison, WI) and quantitative real time PCR was performed by using the UltraSYBR Mixture (CWBIO, China). β-Actin were used as an endogenous control. The primers were as follows: MAPK6-F (realtime) (5′-ACTTGGTGCTGAAGATAG-3′); MAPK6-R (realtime) (5′-TGAGAAGCTCCTGACGAT-3′); β-Actin-F (5′-CCTTCTACAAATGAGCTGCGT-3′); β-Actin-R (5′-CCTGGATAGCAACGTA CATG-3′). All samples were normalized to internal controls and fold changes were calculated through relative quantification (2^−ΔΔct^).

### Western blot

For protein extraction, cells were homogenized on ice in RIPA buffer (Beyotime, China) with PMSF (Beyotime, China) and cellular debris was pelleted at 12,000 g for 30 min at 4°C. Protein was quantified by using enhanced BCA Protein Assay Kit (Beyotime, China) and separated by 8% sodium dodecyl sulfate-polyacrylamide gel (SDS-PAGE), and then transferred to PVDF membranes followed by blocking with 5% non-fat milk. The membranes were incubated with primary antibody against MAPK6 pAb (Bioworld, China), followed by HRP-conjugated secondary antibody (goat anti-rabbit antibody and goat anti-mouse antibody, ZSGB-BIO, China). The membranes were then washed and detection was performed by using the enhanced chemiluminescence western blot detection system (Millipore, USA). Images of each band were analyzed by Bio-Rad Gel Imaging System and normalized to β-actin mAb (Beijing Com Win Biotech Co. Ltd, China). Representative images of three independent experiments are shown in the results section.

### Luciferase assay

In order to assay the effect of HBV on miR499a promoter, the pCH9/3091, pCMV-Sport6-HBx, pCMV-Sport6-HBs, pCMV-Sport6-HBc, and pCMV-Sport6-HBp plasmids were relatively cotransfected with pGL3-Basic-miR499a and pRL-TK into SMMC-7721 cells. In order to assay the effect of miR499a on the 3′UTR of MAPK6, SMMC-7721 cells were cotransfected with pGL3-control-MAPK6-wt or pGL3-control-MAPK6-mut and pTarget-miR499a. Each sample was also cotransfected with pRL-TK. Cells were harvested 48 h later and assayed with the Dual-luciferase Reporter Assay System (Promega, Madison, WI). Relative luciferase activity was normalized to renilla luciferase activity. Transfections were done in duplicate and repeated at least 3 times in independent experiments.

### Cell proliferation assay and colony formation assay

After 24 h of transfection, cells were trypsinized and plated into 96-well plates (8000 per well). At different time points (12 h, 24 h, 48 h), cells were determined by using the MTS kits (Promega, WI, USA) following the manufacturer's protocol and the absorption was read at 490 nm. For colony formation assay, cells were trypsinized and plated in 6-well plates (2000 per well) after transfected for 24 h. Colonies were counted 14 days later using Crystal violet fixed.

### Analysis of tumorigenicity in nude mice

This study was carried out in strict accordance with the recommendations in the Guide for the Care and Use of Laboratory Animals of the National Institutes of Health. The protocol was approved by the Committee on the Ethics of Animal Experiments of the Chongqing Medical University. All surgery was performed under sodium pentobarbital anesthesia, and all efforts were made to minimize suffering. Male BALB/c nude mouse mice aged 4 weeks were used for human tumor xenograft model (supplied by the Chongqing Medical University Animal Center, chongqing, China). s-pTarget or s-pmiR499a cells (5×10^6^) were mixing in 200 µl MEM medium and then injected subcutaneously into the posterior flank of nude mice respectively. Tumor growth was examined every week. Tumor volume (V) was monitored by measuring the length (L) and width (W) with calipers and calculated with the formula (L × W) ×0.5.

### Wound healing assay

SMMC-7721 cells transfected with pTarget or pTarget-miR499a were seeded in 2 ml of RPMI 1640 medium without fetal bovine serum. After 12 h, cells were wounded by dragging a 200 µl pipette tip through the monolayer. PBS was used to wash cellular debris for 3 times and cells were cultured in RPMI 1640 medium with 2% fetal bovine serum. Cell migration images were photographed by using microscope when the scrape wound was introduced (0 h) and at a designated time 24 h and 48 h. The dividing the length of the gap by the culture time showed the speed of migration. Three replicates each of two independent experiments were performed

### Transwell assay

The migration ability of the cells was assessed using Transwell Chambers (8 mm pore size; Millipore). SMMC-7721 cells were severally transfected with pTarget, pTarget-miR499a, NC, miR499a inhibitor, pCH9 or pCH9/3091. After 24 h transfection, cells were resuspended in 5% serum medium. 200 µl of the single-cell suspension (1×10^4^, per well) was seeded onto the upper chamber of each transwell, and the lower 24-well chamber was filled with 600 µl of the cell culture medium supplemented with 10% FBS as a chemo-attractant. A sterile cotton swab was used to remove the cells that did not migrate from the upper surface of the membranes. The membranes were fixed with 4% formaldehyde and stained with 0.05% crystal violet. Then the cells adhered to the lower surface of the membranes were photographed and counted by using microscope(100×) in three randomly selected areas per well.

### Statistical analysis

All the data which was expressed as the mean ± SD was repeated at least thrice. T test was used for analyzed the difference between the control and experimental test. The value of *p*<0.05 was considered statistically significant.

## Supporting Information

Figure S1 **RT-PCR analysis of HBV four expression plasmid in HepG2 cells.** pCMV-Sport6-HBx, pCMV-Sport6-HBs, pCMV-Sport6-HBc and pCMV-Sport6-HBp were transferred into HepG2 cells, respectively. Then RNAs were extracted, digested with DNase I and reverse transcripted into cDNAs. 1. pCH-9/3091 plasmid used as positive control. 2, cDNA reverse transcripted from RNA of HepG2 cells. 3, cDNA reverse transcripted from RNA of HepG2 cells which transferred 1 µg plasmid. 4, cDNA reverse transcripted from RNA of HepG2 cells which transferred 3 µg plasmid. (HBx primers are not good here).
(TIF)

Figure S2 **HBc expression was checked with Western blotting.** 1, HepG2 cellular proteins transfected with pCMV-Sport6-HBc. 2, HepG2.2.15 cellular proteins (positive control). 3, HepG2 cellular proteins (negative control).
(TIF)

## References

[1] Seeff LB, Hoofnagle JH (2006) Epidemiology of hepatocellular carcinoma in areas of low hepatitis B and hepatitis C endemicity. Oncogene 25: 3771–3777. 16799618 10.1038/sj.onc.1209560

[2] Kew MC (2010) Epidemiology of hepatitis B virus infection, hepatocellular carcinoma, and hepatitis B virus-induced hepatocellular carcinoma. Pathol Biol 58: 273–277. 20378277 10.1016/j.patbio.2010.01.005

[3] Beasley RP, Hwang LY, Lin CC, Chien CS (1981) Hepatocellular carcinoma and hepatitis B virus. A prospective study of 22707 men in Taiwan. Lancet 2: 1129–1133. 6118576 10.1016/s0140-6736(81)90585-7

[4] Arbuthnot P, Kew M (2001) Hepatitis B virus and hepatocellular carcinoma. Int J Exp Pathol 82: 77–100. 11454100 10.1111/j.1365-2613.2001.iep0082-0077-x PMC2517704

[5] Pablo L, Mirabela R, Robert S, Alain S, Nicola I, et al (2007) A Mammalian microRNA Expression Atlas Based on Small RNA Library Sequencing. Cell 129: 1401–1414. 17604727 10.1016/j.cell.2007.04.040 PMC2681231

[6] Calin GA, Croce CM (2006) MicroRNA signatures in human cancers. Nat Rev Cancer 6: 857–866. 17060945 10.1038/nrc1997

[7] Lu J, Getz G, Miska EA, Alvarez-Saavedra E, Lamb J, et al (2005) MicroRNA expression profiles classify human cancers. Nature 9: 834–838. 10.1038/nature03702 15944708

[8] Schickel R, Boyerinas B, Park SM (2008) PeterME (2008) MicroRNAs: key players in the immune system, differentiation, tumorigenesis and cell death. Oncogene 27: 5959–5974. 18836476 10.1038/onc.2008.274

[9] Hammond SM (2006) MicroRNAs as oncogenes. Curr OpinGenet 16: 4–9. 10.1016/j.gde.2005.12.005 16361094

[10] Du W, Ma XL, Zhao C, Liu T, Du YL, et al (2014) Associations of Single Nucleotide Polymorphisms in miR-146a, miR-196a, miR-149 and miR-499 with Colorectal Cancer Susceptibility. Asian Pac J Cancer Prev 15(2): 1047–1055. 24568449 10.7314/apjcp.2014.15.2.1047

[11] Li M, Zhang Q, Wu L, Jia C, Shi F, et al (2014) Serum miR-499 as a novel diagnostic and prognostic biomarker in non-small cell lung cancer. Oncol Rep 31(4): 1961–1967. 24549225 10.3892/or.2014.3029

[12] Olivieri F, Antonicelli R, Spazzafumo L, Santini G, Rippo MR, et al (2014) Admission levels of circulating miR-499-5p and risk of death in elderly patients after acute non-ST elevation myocardial infarction. Int J Cardiol 172(2): e276–278. 24461971 10.1016/j.ijcard.2013.12.203

[13] Li X, Wang J, Jia Z, Cui Q, Zhang C, et al (2013) MiR-499 regulates cell proliferation and apoptosis during late-stage cardiac differentiation via Sox6 and cyclin D1. PLoS One 8(9): e74504. 24040263 10.1371/journal.pone.0074504 PMC3770584

[14] Coulombe P, Meloche S (2007) Atypical mitogen-activated protein kinases: structure, regulation and functions. Biochim Biophys Acta 1773(8): 1376–1387. 17161475 10.1016/j.bbamcr.2006.11.001

[15] Déléris P, Rousseau J, Coulombe P, Rodier G, Tanguay PL, et al (2008) Activation loop phosphorylation of the atypical MAP kinases ERK3 and ERK4 is required for binding, activation and cytoplasmic relocalization of MK5. J Cell Physiol 217(3): 778–788. 18720373 10.1002/jcp.21560

[16] Seternes OM, Mikalsen T, Johansen B, Michaelsen E, Armstrong CG, et al (2004) Activation of MK5/PRAK by the atypical MAP kinase ERK3 defines a novel signal transduction pathway. EMBO J 23(24): 4780–4791. 15577943 10.1038/sj.emboj.7600489 PMC535098

[17] Sergiy K, Gianina D, Ugo M (2012) Tumour promoting and suppressing roles of the atypical MAP kinase signalling pathway ERK3/4-MK5. J Molecular Signaling 7(1): 9. 10.1186/1750-2187-7-9 PMC3419095 22800433

[18] Luo J, Deng ZL, Luo X, Tang N, Song WX, et al (2007) A protocol for rapid generation of recombinant adenoviruses using the AdEasy system. Nat Protoc 2: 1236–1247. 17546019 10.1038/nprot.2007.135

[19] Buxade M, Parra-Palau JL, Proud CG (2008) The Mnks: MAPK kinase-interactingkinases (MAP kinase signal-integrating kinases). Front Biosci 13: 5359–5373. 18508592 10.2741/3086

[20] Lai CL, Yuen MF (2008) Chronic hepatitis B–new goals, new treatment. N Engl J Med 359(23): 2488–2491. 19052131 10.1056/NEJMe0808185

[21] Yang HI, Yeh SH, Chen PJ, Iloeje UH, Jen CL, et al (2008) Associations between hepatitis B virus genotype and mutants and the risk of hepatocellular carcinoma. J Natl Cancer Inst 100(16): 1134–1143. 18695135 10.1093/jnci/djn243 PMC2518166

[22] Sengupta S, den Boon JA, Chen IH, Newton MA, Stanhope SA, et al (2008) MicroRNA 29c is down-regulated in nasopharyngeal carcinomas, up-regulating mRNAs encoding extracellular matrix proteins. Proc Natl Acad Sci 105: 5874–5878. 18390668 10.1073/pnas.0801130105 PMC2311339

[23] Lagos-Quintana M, Rauhut R, Lendeckel W, Tuschl T (2001) Identification of novel genes coding for small expressed RNAs. Science 294: 853–858. 11679670 10.1126/science.1064921

[24] Wang Z, Wu J, Zhang G, Cao Y, Jiang C, et al (2013) Associations of miR-499 and miR-34b/c Polymorphisms with Susceptibility to Hepatocellular Carcinoma: An Evidence-Based Evaluation. Gastroenterol Res Pract 2013: 719202. 24194751 10.1155/2013/719202 PMC3804138

