# Supplementary figures and images for: Up-Regulated MicroRNA499a by Hepatitis B Virus Induced Hepatocellular Carcinogenesis via Targeting MAPK6
Source: PLoS One. 2014 Oct 23;9(10):e111410. doi: 10.1371/journal.pone.0111410 (PMC4207808; doi:10.1371/journal.pone.0111410)

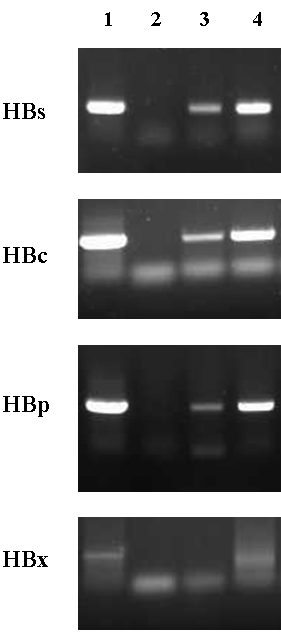

Supplement: Figure S1 — RT-PCR analysis of HBV four expression plasmid in HepG2 cells. pCMV-Sport6-HBx, pCMV-Sport6-HBs, pCMV-Sport6-HBc and pCMV-Sport6-HBp were transferred into HepG2 cells, respectively. Then RNAs were extracted, digested with DNase I and reverse transcripted into cDNAs. 1. pCH-9/3091 plasmid used as positive control. 2, cDNA reverse transcripted from RNA of HepG2 cells. 3, cDNA reverse transcripted from RNA of HepG2 cells which transferred 1 µg plasmid. 4, cDNA reverse transcripted from RNA of HepG2 cells which transferred 3 µg plasmid. (HBx primers are not good here). (TIF) [file pone.0111410.s001.tif]

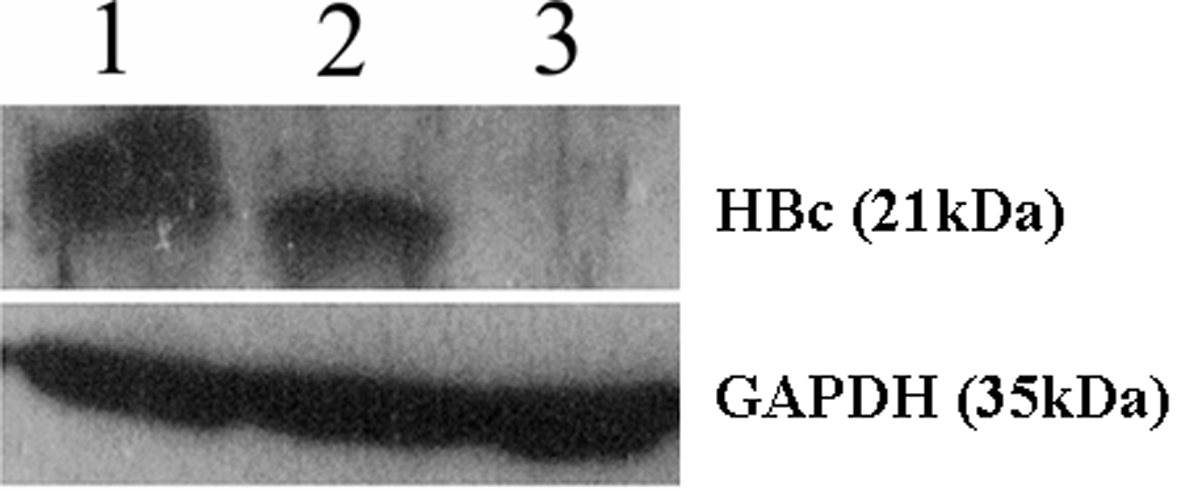

Supplement: Figure S2 — HBc expression was checked with Western blotting. 1, HepG2 cellular proteins transfected with pCMV-Sport6-HBc. 2, HepG2.2.15 cellular proteins (positive control). 3, HepG2 cellular proteins (negative control). (TIF) [file pone.0111410.s002.tif]
